# Expressing acetylcholine receptors after innervation suppresses spontaneous vesicle release and causes muscle fatigue

**DOI:** 10.1038/s41598-017-01900-3

**Published:** 2017-05-10

**Authors:** Meghan Mott, Victor M. Luna, Jee-Young Park, Gerald B. Downes, Kimberly Epley, Fumihito Ono

**Affiliations:** 1 Section on Model Synaptic Systems, Laboratory of Molecular Physiology, NIAAA, NIH, Bethesda, Maryland 20892 USA; 2 Department of Psychiatry, Columbia University, and Division of Integrative Neuroscience, New York State Psychiatric Institute, New York, NY 10032 USA; 3 Biology Department, University of Massachusetts Amherst, Amherst, Massachusetts, 01003 USA; 4 Whitney laboratory for Marine Bioscience, University of Florida, St. Augustine, Florida, 32080 USA; 5 Department of Physiology, Osaka Medical College, Takatsuki, 569–8686 Japan

## Abstract

The formation and function of synapses are tightly orchestrated by the precise timing of expression of specific molecules during development. In this study, we determined how manipulating the timing of expression of postsynaptic acetylcholine receptors (AChRs) impacts presynaptic release by establishing a genetically engineered zebrafish line in which we can freely control the timing of AChR expression in an AChR-less fish background. With the delayed induction of AChR expression after an extensive period of AChR-less development, paralyzed fish displayed a remarkable level of recovery, exhibiting a robust escape response following developmental delay. Despite their apparent behavioral rescue, synapse formation in these fish was significantly altered as a result of delayed AChR expression. Motor neuron innervation determined the sites for AChR clustering, a complete reversal of normal neuromuscular junction (NMJ) development where AChR clustering precedes innervation. Most importantly, among the three modes of presynaptic vesicle release, only the spontaneous release machinery was strongly suppressed in these fish, while evoked vesicle release remained relatively unaffected. Such a specific presynaptic change, which may constitute a part of the compensatory mechanism in response to the absence of postsynaptic AChRs, may underlie symptoms of neuromuscular diseases characterized by reduced AChRs, such as myasthenia gravis.

## Introduction

The vertebrate NMJ is a cholinergic synapse formed between a motor nerve and skeletal muscle. Zebrafish provide an exceptional model system to observe this synapse formation *in vivo* because of their transparency, rapid development, and the various genetic tools available to alter their synaptic function. In zebrafish embryos, neuromuscular synapses start to function and lead to spontaneous locomotion around 17 hours post fertilization (hpf)^1^. We previously analyzed a mutant zebrafish that lacked AChRs in the NMJ and found the morphology of its presynaptic terminals was largely normal^2^. However, our analysis of the functional consequences of losing postsynaptic AChRs on the presynaptic machinery was limited because synaptic currents were not measurable^3^.

In order to address this issue, here we have established a genetically engineered zebrafish line in which we can freely control the timing of AChR expression in an AChR-less fish background using a chemically inducible gene expression system. By allowing a sufficient time lag before inducing the expression of AChRs, we could observe the effect of AChR-less development on NMJ synaptic currents. We found that these synapses exhibited remarkable adaptability, which inevitably led to functional transmission. However, these rescued synapses manifested characteristics that were remarkably distinct from their normally developed counterparts, namely the lack of spontaneous vesicle releases. These differences impacted their swimming performance.

## Results

**Figure 1:**
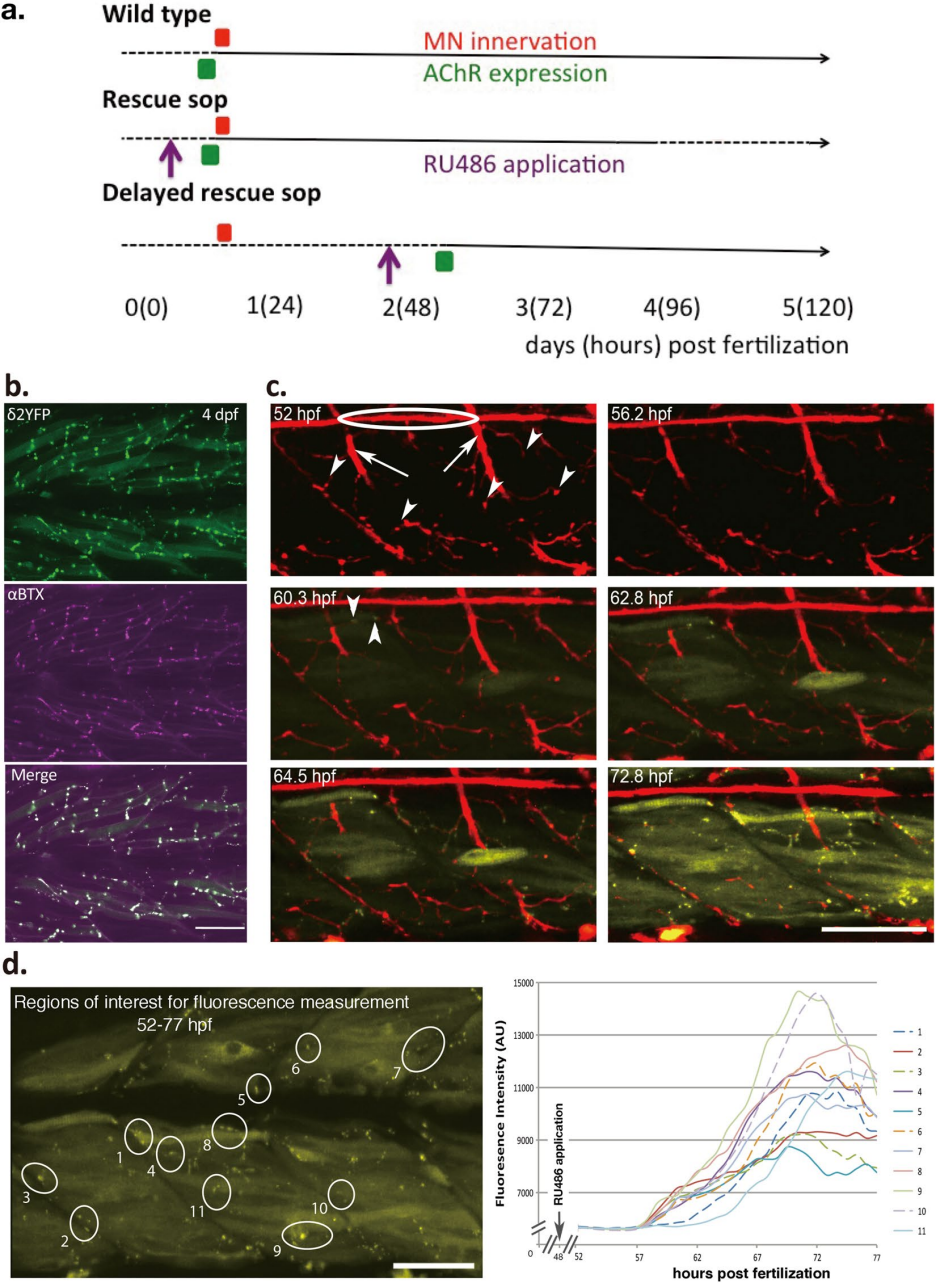
Synapse formation in *delayed rescue sop*. (**a**) A summary of the experimental paradigm, showing events related to the synapse development and manipulations along the developmental time course. Numbers indicate days post fertilization (hours post fertilization in parentheses). Dashed lines indicate periods when zebrafish embryos are immotile, and solid lines indicate stages when embryos exhibit mobility. Timing of motor neuron axon innervation (red), AChR expression (green) and RU486 application (purple) are indicated by boxes and arrows. (**b**) δ2YFP (top), αBTX (middle) and merged (bottom) in the trunk region of a 4 dpf *delayed rescue sop* are shown. (**c**) Time-lapse images of axons (red) and AChRs (yellow) *in vivo* from *delayed rescue sop* embryos. In the 52 hpf panel, lateral line axons (circled) and motor neuron trunks (arrows) are visualized. Motor neuron terminals are indicated by arrowheads. In the 60.3 hpf panel, the first expressed AChR clusters are detected, indicated by arrowheads. (**d**) Analysis of fluorescence intensity of identified regions with AChR clusters in *delayed rescue sop*. Fluorescence from the marked regions of interest (labeled 1–10) was measured and plotted against hours post fertilization. RU486 was applied at 48 hpf, before the measurement of fluorescence started. Scale bars: 50 µm.

We generated a transgenic zebrafish line in which the externally applied chemical RU486 controls the temporal expression of AChRs^4^ (Supplementary Fig. S1a). This chemically inducible system was crossed into *sofa potato* (*sop*), a paralyzed mutant zebrafish line lacking postsynaptic AChRs in the NMJ^5^. Induction by RU486 at either ~10 hpf or ~48 hpf led to the expression of AChRs in *sop*, detectable by the YFP-tagged δ subunit (δ2YFP)^6^ (Fig. 1b).

Treatment with RU486 at ~10 hpf led to movement of *rescue sop* (Fig. 1a, middle) that were indistinguishable from their wild type siblings (Fig. 1a, top), until the induced protein degenerated and disappeared after 3 dpf. This is in agreement with our previous study, in which AChR gene expression was driven directly by the α1a-actin (α-actin) promoter in the *sofa potato* background. *Sop* fish expressing δ2YFP mounted a normal escape response^6^. In contrast, when RU486 was applied later in development at ~48 hpf, this led to a delayed expression of AChRs at ~60 hpf, allowing an extended period of paralyzed development (Fig. 1a, bottom). We will refer to these fish as “*delayed rescue sop*” (Fig. 1a, bottom).

**Figure 2:**
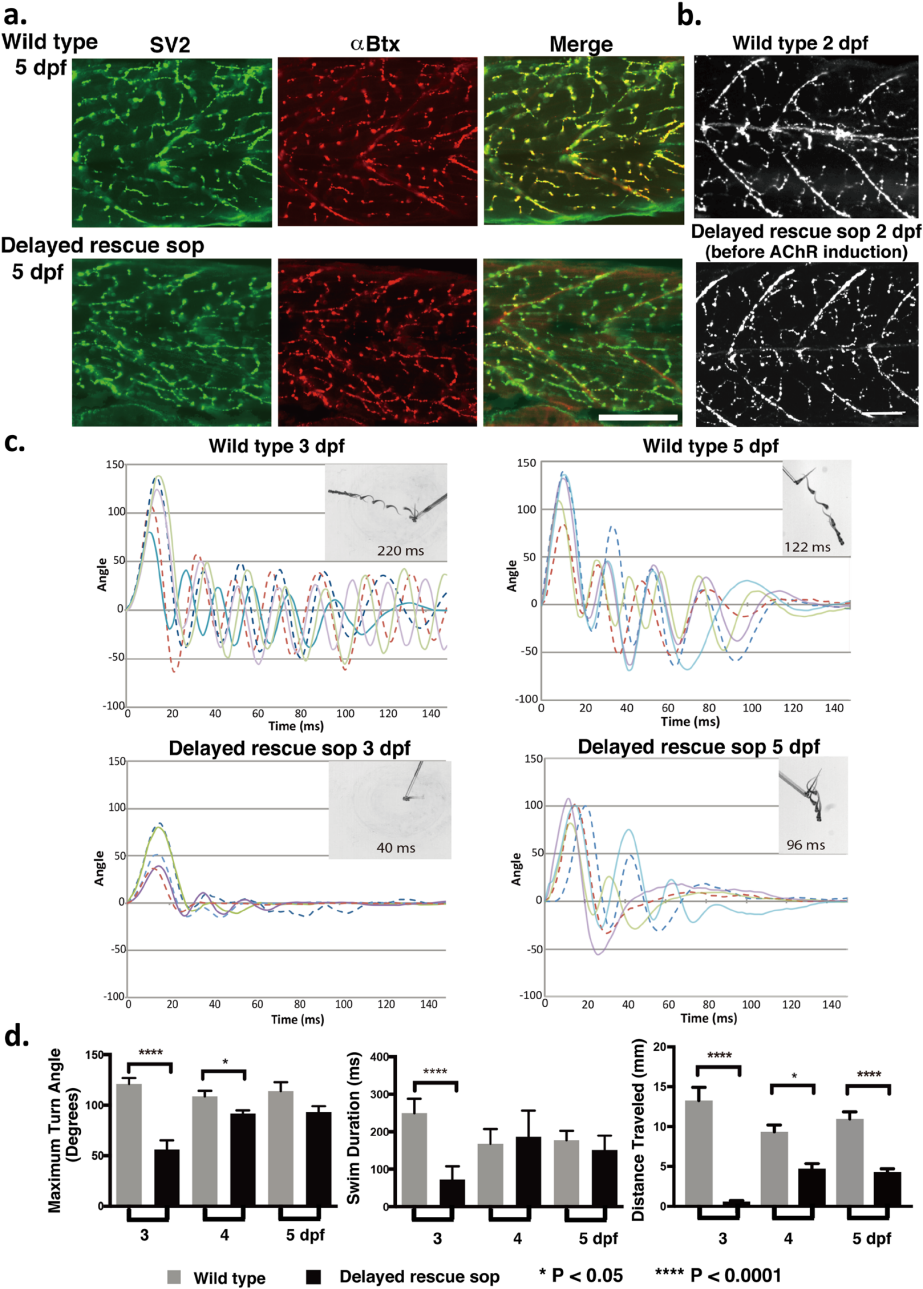
Locomotion of *delayed rescue sop*. (**a**) Nerve terminals visualized by anti-SV2 antibody that overlap with the postsynaptic AChRs visualized by αBTX, in wild type and *delayed rescued sop* embryos at 5 dpf. Scale bar, 50 µm. (**b**) anti-SV2 antibody staining at 2 dpf, in wild type and in *delayed rescue sop* before AChR induction. (**c**) Escape behaviors in wild type and *delayed rescue sop* at 3 and 5 dpf in response to tactile stimuli. Kinematics for representative traces of 5 larvae are shown for the initial 150 ms of response. Each trace represents a different larva. Body angles are shown in degrees with 0 indicating a straight body, and positive and negative values indicating body bends in opposite directions. Inlaid images of representative larva for each group show superimposed frames of complete escape response and indicate the duration of movement in ms. (**d**) Maximum turn angle, swim duration and distance traveled were calculated for each group of fish (n = 10 per group) at 3, 4, and 5 dpf. Maximum turn angle is defined as the strongest body angle the larva makes in its initial turn (C-bend) away from the stimulus. Swim duration is defined as the length of time the larva swims following the stimulus until returning to a resting state. Distance traveled is defined as the distance between locations of the head at the start and the end of the swimming.

*Delayed rescue sop* were completely paralyzed until their treatment in RU486 at 2 days post fertilization (dpf). At 3 dpf, ~24 hours after RU486 treatment, *delayed rescue sop* fish exhibited a robust response to tactile stimuli (Fig. 2c). However, a clear behavioral difference from control larvae was still observed (Fig. 2c and d). In *delayed rescue sop* fish, the initial turn was significantly less pronounced (120 ± 6° in wild type and 56 ± 9° in *delayed rescue sop*; p < 0.0001) and their subsequent movement did not continue beyond the second body bend. At 3 dpf, the swim duration was 249 ± 12 ms for wild type and 72 ± 13 ms for *delayed rescue sop* (p < 0.0001). Interestingly, at 5 dpf the swimming of *delayed rescue sop* was markedly improved (Fig. 2c). The differences in maximum turn angle and swim duration were no longer observed between *delayed rescue sop* and wild type fish (Fig. 2d). However, the distance traveled was still shorter in *delayed rescue sop*: 11.0 ± 0.9 mm for wild type and 4.3 ± 0.4 mm for *delayed rescue sop* (p < 0.0001; Fig. 2d). The shorter distance traveled in *delayed rescue sop* is likely a result of the weaker body bends following the initial strong turn (Fig. 2c). To determine how these swimming behaviors correlated with the anatomical development of neuromuscular synapses, we used transgenic fish with fluorescently labeled synaptic proteins.

We crossed the *sop* line carrying the inducible δ2YFP gene with a transgenic line expressing mCherry in motor neurons (MNs)^7^. Using YFP-tagged AChRs and mCherry signals, we examined how the contact between nerve terminals and AChR clusters develops in *delayed rescue sop*. Surprisingly, we found that newly expressed AChRs followed the guidance of motor neuron axons and formed clusters at sites predetermined by nerve terminals (Fig. 1c; For the time lapse movie, see Supplementary Video S2). This is in stark contrast to normal NMJ development^8^ where AChRs form clusters before motor nerve terminals come into contact with them^9^. Therefore, the synapse formation sequence is ostensibly reversed in *delayed rescue sop*.

The clustering of AChRs in *delayed rescue sop* started within 2 hrs after their cytoplasmic expression was initially detected, with receptor density reaching a plateau within 20 hrs (Fig. 1d). Nerve terminals in *delayed rescue sop* displayed positive signals for SV2, a pre-synaptic vesicle marker, which overlapped with post-synaptic receptors at 5 dpf (Fig. 2a). These SV2 signals were observable even before the expression of AChRs, and their distribution was similar to that exhibited in wild type fish (Fig. 2b). This finding agrees with a previous report using FM1-43 to visualize synaptic vesicles^3^. These findings demonstrate that the morphological characteristics of NMJs, such as the AChR clustering and the synaptic vesicle accumulation, were well developed at 3 dpf in *delayed rescue sop*. However, our behavioral findings indicate that synapses needed 2 more days for functional maturation in order for *delayed rescue sop* to mount a vigorous escape response (Fig. 2c,d).

**Figure 3:**
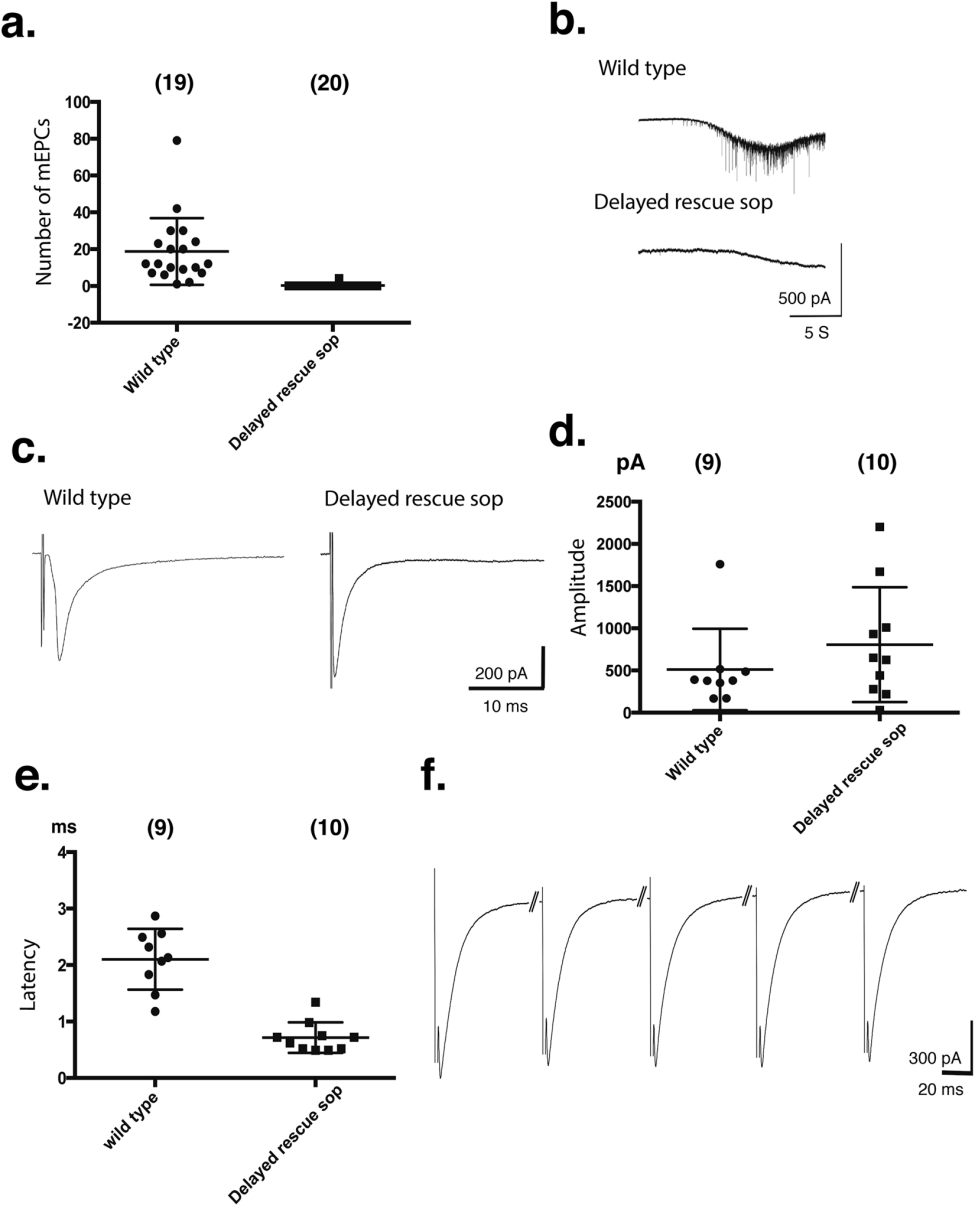
Synaptic functions of *delayed rescue sop* at 4–6 dpf. (**a**) Number of mEPCs recorded from muscle cells of wild type or *delayed rescue sop* in 30 sec. Each point represents a muscle cell. (**b**) mEPCs induced by the application of 0.5 M sucrose. When the hypertonic solution hit the muscle cell, the base line drifted downward, presumably due to the muscle twitching. Scale: 5 S, 500 pA. (**c**) Evoked synaptic currents recorded from wild type and *delayed rescue sop*. Scale: 10 ms, 200 pA. The amplitudes (**d**) and the latency (**e**) of evoked synaptic currents were plotted for each muscle cell. (**f**) Evoked synaptic currents in *delayed rescue sop* with the 20 Hz motor neuron stimulation. Scale: 20 ms, 300 pA.

In order to gain insight into the synaptic physiology of *delayed rescue sop*, we recorded spontaneous synaptic currents using whole-cell patch clamp of skeletal muscle cells. Remarkably, *delayed rescue sop* revealed almost a complete lack of miniature endplate currents (mEPCs) at 4–6 dpf (Fig. 3a). While recordings of wild type muscle cells exhibited up to 80 mEPCs in 30 sec recordings, the majority of muscle cells in *delayed rescue sop* showed no mEPCs and only 1 cell in 20 cells showed 4 mEPCs. The frequency of mEPCs recorded in wild type fish (n = 19) and *delayed rescue sop* (n = 20) was 0.62 ± 0.14 Hz and 0.007 ± 0.007 Hz, respectively. To increase the probability of observing mEPCs, we bath-applied hypertonic solution that induced more robust release of synaptic vesicles. Application of hypertonic solution causes the release of vesicles in the readily releasable pool independent of the intracellular calcium and the action potentials, presumably by mechanically lowering the energy barrier for vesicle fusion^10, 11^. Muscle cells from wild type (n = 8) fish showed a marked increase in mEPCs in response to 0.5 M sucrose (Fig. 3b) while *delayed rescue sop* still failed to show mEPCs (n = 13) (Fig. 3b).

We examined whether evoked synaptic currents were also affected in *delayed rescue sop*. Evoked currents are entirely dependent on motor neuron action potential (AP) firing, in contrast to mEPCs which result from spontaneous AP-independent release of synaptic vesicles^12^. The average amplitude of evoked currents from *delayed rescue sop* (n = 10) was not significantly different from that of their wild type siblings (n = 9) (Fig. 3c,d; 511 ± 161 and 805 ± 215 pA; p = 0.35). However, the latency after stimulation was shorter in *delayed rescue sop*: 2.10 ± 0.18 vs 0.72 ± 0.08 ms (p = 0.053; Fig. 3e). We also recorded evoked synaptic responses in *delayed rescue sop* induced by 20 Hz stimulation of motor neurons. The amplitudes of these evoked currents did not decrease with repeated stimuli (Fig. 3f), suggesting that the replenishment of the vesicle pool is retained. Taken together, these data suggest that the change in *delayed rescue sop* synaptic function was specific to spontaneous vesicle release.

## Discussion

The *delayed rescue sop* fish established in this study are the first NMJ mutant in which the synapse formation sequence is reversed such that the innervation of motor neuron axons precedes that of postsynaptic AChR expression (Fig. 1). Analyses of other molecules involved in the NMJ formation will provide further insights of signaling cascades. For example, MuSK-Lrp4 complex, which forms pre-clusters at NMJs preceding clusters of AChRs^13^, may guide the axon terminals in *delayed rescue sop*. Here we took advantage of the unique opportunity provided by the *delayed rescue sop* system to elucidate how the precise timing of molecular events contributes to normal and aberrant neuromuscular synaptic formation, physiology, and behavior.

Remarkably, we found that synapses that eventually form in *delayed rescue sop* largely lack spontaneous vesicle release. This finding is consistent with previous studies showing that inhibition of postsynaptic receptors result in the compensatory increase of quantal contents and reduced spontaneous vesicle release^14^.

NMJs in myasthenia gravis, an autoimmune disease in which AChRs are attacked by antibodies and degraded, have reduced density of AChRs^15^. Presynaptic compensation manifested as an increase in quantal content has been shown to offset this reduction of AChRs^16^. The reduction of spontaneous vesicle release is also observed in parallel. Although its significance *per se* is not clear, it may also constitute a part of the presynaptic compensation. Pre-synaptic changes are also observed in NMJs with reduced functional receptors in rats, mice and fruit flies^17–19^. In comparison to these other studies, *delayed rescue sop* fish manifested a much stronger phenotype and, interestingly, were selectively impaired in spontaneous synaptic transmission.

In addition to presynaptic phenotypes, *delayed rescue sop* showed muscle fatigue with normal strength of the initial turn followed by weaker body bends (Fig. 2c and d). Muscle fatigue is also characteristic of human diseases affecting AChRs in NMJs, such as myasthenia gravis^15^. We speculate that presynaptic terminals in *delayed rescue sop* compensate for the lack of postsynaptic responses earlier in development (up to 3–4 dpf) and that the effect remains even after the normal density of AChRs is achieved at ~5 dpf. Thus compensatory mechanisms akin to myasthenia gravis may be responsible for the *delayed rescue sop* phenotypes.

Evoked synaptic currents in *delayed rescue sop* were similar to wild type larvae in amplitude, but were different in kinetics, most notably in latency (Fig. 3c–e). Evoked synaptic currents are linked to locomotion more directly than mEPCs, and may underlie the changes in swimming observed in *delayed rescue sop* (Fig. 2c). These differences in kinetics may arise from several possible mechanisms, such as differences in membrane excitability or the dynamics of vesicle release regulated by intracellular calcium. However, further analysis is limited in the extracellular stimulation method we employed to evoke synaptic currents because multiple motor neurons are recruited, not necessarily simultaneously, by a single stimulus. Future studies employing double patch clamp of a motor neuron and a muscle cell as well as electron microscopy will shed light on the mechanisms underlying the synaptic changes observed in *delayed rescue sop* ^20^.

The *delayed rescue sop* provides an ideal framework for isolating and investigating molecular factors that lead to the specific suppression of spontaneous vesicle release, an area of research that has recently attracted much attention^21^. The physiological role of spontaneous vesicle release has been the subject of controversy for many years, but recent studies have suggested that its function may be distinct from evoked neurotransmission in synapse development^19^. Most importantly, identification of these factors could aid in alleviating some of the more debilitating symptoms of patients suffering from reduced AChRs in NMJs like myasthenia gravis.

## Methods

### Fish lines

Zebrafish were maintained in the self-circulating systems at NIAAA/NIH and the Osaka Medical College. Transgenic fish, tg(α-actin: δ2YFP) and tg(HuC: mCherry), were crossed with *sofa potato* mutants (*sop* tj^95*d*^)^22^. All methods were carried out in accordance with relevant guidelines and regulations. All experimental protocols were approved by IACUCs at NIAAA/NIH and the Osaka Medical College.

### Construct and chemical induction of receptors

A DNA construct containing the α-actin promoter driving LexA^DBD^ + PR^LBD^ + p65^AD^ and the LexA operator fused to the minimal 35 S promoter driving the δ2YFP expression was constructed by using Gateway system (Supplementary Fig. S1). The construct had Tol2 sequence for efficient integration, and a stable line was established following a method described earlier^23^. Embryos injected with the DNA construct and transposase at the 1 cell stage were raised to adult, and out-crossed to search for germ-line transmission of the transgene, using the expression of GFP in response to RU486 for screening. The established stable line was crossed to mutants/transgenic lines for experiments.

RU486, also called mifepristone, is a progesterone antagonist, and can bind to the LexPR transactivator (LexA^DBD^ + PR^LBD^ + p65^AD^) and induce the expression of δ2YFP, which encodes the AChR δ subunit tagged with YFP in the cytoplasmic loop^6^. The induction with RU486 for *delayed rescue sop* was performed with ~20 min application of 100 nM–1 μM RU486 with minimal side effects. For the *rescue sop* experiment, embryos at ~10 hpf were treated with RU486 for longer periods (3–12 hours), because the exact timing of the LexPR transactivator expression driven by the α-actin promoter was difficult to determine. The side effect was again not noticeable. Wild type controls were treated with RU486 in parallel for each experiment.

### Immunohistochemistry and confocal microscopy

Immunohistochemistry was performed as previously described^24^. Briefly, larvae were fixed with 4% paraformaldehyde at 4 °C. The fish were washed with distilled water for 5 min, treated with acetone for 7 min at −20 °C, and thoroughly washed again with water. After incubation in PBS containing 2% horse serum and 0.5% Triton X-100, the fish were treated with the SV2 antibody (1:500, the Developmental Studies Hybridoma Bank at the University of Iowa, cat# SV2)^22^. After thorough washing, the fish were incubated with a secondary antibody (Goat anti-mouse Alexa488 conjugated antibody, 1:500, Invitrogen cat# A11001). After another round of thorough washing, samples were mounted on glass-bottom petri dish and observed under the confocal microscope. For labeling with α-Bungarotoxin (αBtx), beheaded larvae without or after fixation was treated with 10^−6^ M αBtx for 20 min and washed thoroughly. Confocal imaging including the time-lapse study was performed on Zeiss 510 Meta. Obtained images on the Zeiss ZEN software were transferred to Photoshop and processed further. For time-lapse study, developing larvae anaesthetized in Tricaine were embedded in 1.2% low melting temperature agarose and sequential images were captured on Zeiss 510 Meta.

### Swimming analysis

Swimming of larvae at 3–5 dpf was recorded with Photron high-speed camera at 1000 frames/sec. Gentle touch to the head with a von Frey filament standardized at 4 grams of force was used to stimulate escape behaviors. Obtained images were analyzed off line with a custom built semi-automatic kinematic analysis software, which measures the head-to-tail angle for each frame of the response^25^. The frame at 0 ms was chosen before the larval movement was first detected. Measured angles were plotted against time (Fig. 2c).

### Electrophysiology

Recording of synaptic currents in the NMJ as well as the extracellular stimulation of motor neurons was performed as previously described with some modifications^2, 26^. Skinned larvae were pinned down to the recording chamber coated with Sylgard and immobilized either by treatment with 2 M formamide for 5 min or bathing in 10 μM nifedipine^27^. Patch-clamp recordings were made by the whole-cell ruptured technique of muscle cells. The pipette solution used for the voltage-clamp recording was (in mM) 120 KCl, 5 BAPTA, and 5 HEPES, pH 7.2. The extracellular solution contained (in mM) 112 NaCl, 2 KCl, 2 CaCl_2_, 1 MgCl_2_, 3 glucose, and 5 HEPES, pH 7.4. Membrane currents were recorded with an EPC10 amplifier and PatchMaster. For mEPC recordings, muscle cells were voltage clamped at −90 mV and 1 μM TTX was added to the recording solution. The currents were sampled at 50 kHz and filtered at 3–5 kHz before analysis. Capacitive transients were compensated by manual compensation.

To stimulate the spinal cord for evoked synaptic current recordings, platinum wire electrodes were pressed on the body surface from above and below the fish at the level of spinal cord. Spinal neurons were stimulated with an isolated pulse stimulator (A-M Systems, Model 2100), and the stimulus strength was adjusted to the lowest voltage that consistently evoked synaptic responses in muscle. The muscle cell was voltage clamped at −50 mV, to desensitize Na^+^ channels and avoid the direct channel activation by the extracellular stimulation^26^.

The application of hypertonic sucrose solution was performed by two methods. One method used a glass electrode filled with the bath solution containing 0.5 M sucrose placed near the recording electrode, and a positive air pressure was applied through Picospritzer II. The other method used a peristaltic pump to replace the bath solution with the sucrose solution. mEPCs in *delayed rescue sop* were not observed in either method.

### Statistics

Unpaired t-test (two tailed) was performed for statistical analysis on Prism. Averages and standard errors of the mean are displayed. Numbers of samples are shown in parentheses.

## Electronic supplementary material

Supplementary Figure S1

Supplementary Video S2
