# Supplementary material for: Expressing acetylcholine receptors after innervation suppresses spontaneous vesicle release and causes muscle fatigue
Source: Sci Rep. 2017 May 10;7:1674. doi: 10.1038/s41598-017-01900-3 (PMC5431962; doi:10.1038/s41598-017-01900-3)
Supplement: Supplementary file 1 — Supplementary Figure S1 [file 41598_2017_1900_MOESM1_ESM.doc]

**Expressing acetylcholine receptors after innervation suppresses spontaneous vesicle release and causes muscle fatigue**


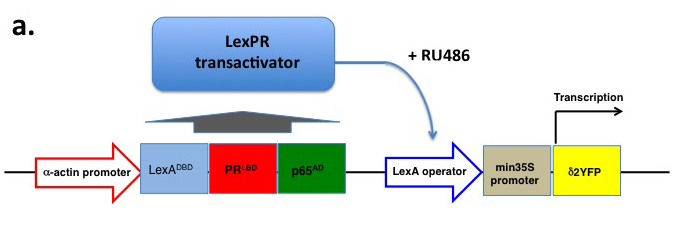
Meghan Mott, Victor M. Luna, Jee-Young Park, Gerald B. Downes, Kimberly Epley, Fumihito Ono


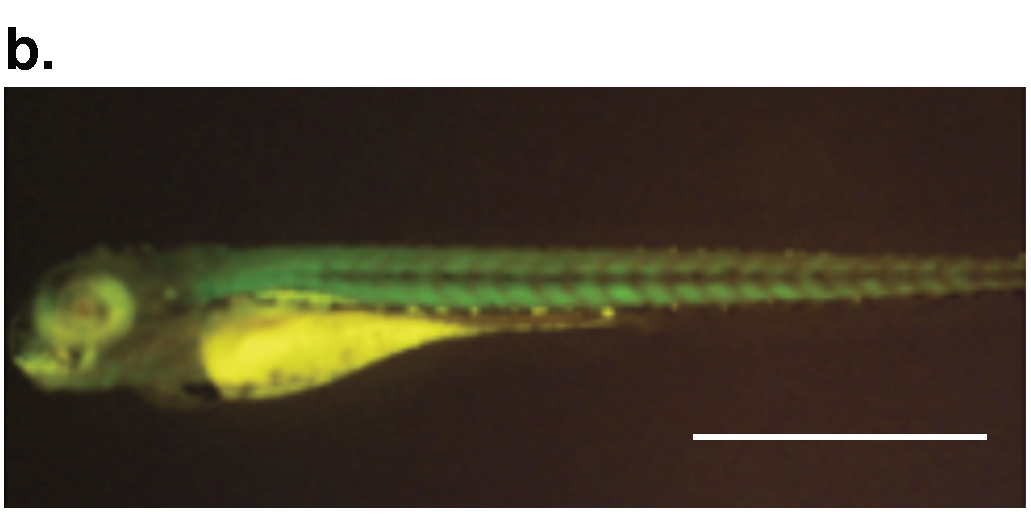


**Supplementary Figure S1** Induction of AChR in muscle cells by RU486.

***a,*** The scheme of the DNA construct employed for the RU486-inducible system. α-actin promoter drives the expression of a fusion protein^4^: LexA^DBD^+PR^LBD^+p65^AD^. LexA^DBD^ is a DNA-binding region of the bacterial LexA protein. PR^LBD^ is a ligand binding domain of the human progesterone receptor. p65^AD^ is the activation domain of the human p65. The δ2YFP gene is downstream of the LexA operator fused to the minimal 35S promoter from Cauliflower Mosaic Virus^4^. When RU486 binds to the fusion protein, it binds to the LexA operator and drives the expression of δ2YFP. ***b,*** The expression of AChRs induced by the RU-486 application. YFP tagged to the AChR was observed in the trunk muscle region. Scale: 1 mm.
